# Epidemiology and Molecular Characterisation of Multidrug-Resistant *Escherichia coli* Isolated from Cow Milk

**DOI:** 10.3390/vetsci11120609

**Published:** 2024-11-29

**Authors:** Zarin Tasnim Mim, Chandan Nath, Abdullah Al Sattar, Rijwana Rashid, Mehedy Hasan Abir, Shahneaz Ali Khan, Md Abul Kalam, Shahanaj Shano, Rowland Cobbold, John I. Alawneh, Mohammad Mahmudul Hassan

**Affiliations:** 1 Faculty of Veterinary Medicine, Chattogram Veterinary and Animal Sciences University, Chattogram 4225, Bangladesh; zarintasnimmim.1bd@gmail.com (Z.T.M.); chandannath227@gmail.com (C.N.); rijwanarashid@gmail.com (R.R.); abir.fst9@gmail.com (M.H.A.); shahneaz@cvasu.ac.bd (S.A.K.); 2 Faculty of Life Sciences: Food, Nutrition and Health, University of Bayreuth, 95447 Bayreuth, Germany; 3 School of Psychology, University of New England, Armidale, NSW 2350, Australia; asattar2@myune.edu.au; 4 Nutritional Sciences Graduate Program, Margaret Ritchie School of Family and Consumer Sciences, College of Agricultural & Life Sciences, University of Idaho, Moscow, ID 83844, USA; 5 Global Health and Development Program, Laney Graduate School, Emory University, Atlanta, GA 30322, USA; shahanaj.shano@emory.edu; 6 Faculty of Science and Engineering, Southern Cross University, East Lismore, NSW 2480, Australia; rowland.cobbold@scu.edu.au; 7 Plant Biosecurity and Product Integrity, Biosecurity Queensland, Department of Agriculture and Fisheries, Brisbane, QLD 4000, Australia; john.alawneh@daf.qld.gov.au; 8 Queensland Alliance for One Health Sciences, School of Veterinary Science, The University of Queensland, Gatton, QLD 4343, Australia

**Keywords:** raw cow milk, *E. coli*, antimicrobial resistance, multidrug resistance, public health

## Abstract

Antimicrobial resistance (AMR) is a growing global concern and poses a significant threat to public health. The emergence of multidrug-resistant organisms, including *Escherichia coli*, also presents a risk of transmission to humans through the food chain, including milk. This study aimed to investigate the prevalence of *E. coli* in raw milk in the Chattogram metropolitan area (CMA) of Bangladesh and their phenotypic and genotypic antimicrobial resistance patterns. A total of 450 raw cow milk samples were collected from 18 farms within the CMA. The isolation and identification of *E. coli* were performed following standard bacteriological methods. Antimicrobial susceptibility testing (AST) was conducted using the Kirby–Bauer disc diffusion method. Molecular detection of *E. coli* and antimicrobial resistance genes was performed using the Polymerase Chain Reaction (PCR). This study found 134 (29.77%) milk samples that tested positive for *E. coli*. Antimicrobial susceptibility testing (AST) revealed the highest resistance rates (69.40%) to be for ampicillin, amoxicillin–clavulanic acid, cephalothin, and cephalexin, with the lowest resistance (21.64%) being for norfloxacin. A significant correlation (*r* = 1) was observed between ciprofloxacin and ceftazidime resistance among the antimicrobials tested. All *E. coli* isolates were classified as multidrug-resistant (MDR), being resistant to three or more antimicrobial classes, with a multiple resistance index >0.2. PCR amplification showed that the *bla*_TEM_ gene had the highest prevalence (74.19%) among the ESBL and antimicrobial resistance genes tested. In contrast, the *bla*_CMY-1_ gene had a lower prevalence (6.45%) among the ESBL genes, while the *tet*D gene had the lowest prevalence (2.9%) among the resistance genes tested. Positive correlations were observed between antimicrobial resistance and the presence of these resistance genes. This study emphasises the high prevalence of MDR *E. coli* in raw cow milk and its significant potential impact on public health. It underscores the urgent need for strategic interventions to effectively manage and mitigate AMR in the Bangladeshi dairy sector, focusing on the prudent use of antimicrobials and implementing enhanced AMR surveillance.

## Simple Summary

Antimicrobial resistance (AMR) is a growing global issue that poses serious public health risks. This study investigated the prevalence and pattern of antimicrobial resistance of *Escherichia coli* in raw cow milk from 18 farms in Chattogram, Bangladesh. Out of 450 samples, 134 (29.77%) tested positive for *E. coli*. Antimicrobial susceptibility testing revealed high resistance rates (69.40%) to ampicillin, amoxicillin–clavulanic acid, cephalothin, and cephalexin, while resistance to norfloxacin was lowest (21.64%). All isolates were multidrug-resistant (MDR), showing resistance to three or more antimicrobial classes, with a multiple resistance index >0.2. PCR testing detected the *bla*_TEM_ gene in 74.19% of isolates, the highest among extended-spectrum beta-lactamase (ESBL) genes. The *bla*_CMY-1_ gene was less prevalent (6.45%), and the *tet*D gene was rare (2.9%). Positive correlations were noted between antimicrobial resistance and resistance gene presence, with a strong link (*r* = 1) between ciprofloxacin and ceftazidime resistance. This study highlights the significant presence of MDR *E. coli* in raw milk, posing a potential public health threat through the food chain. It calls for urgent measures to manage AMR, including prudent antimicrobial use, enhanced surveillance, and targeted interventions in Bangladesh’s dairy sector.

## 1. Introduction

Antimicrobial resistance (AMR) is a major global health threat that diminishes the effectiveness of bacterial disease treatments, leading to more complex, prolonged, costly, and difficult healthcare interventions. It is estimated that AMR may result in an extra 10 million deaths per year, 100 trillion USD in economic cost, and an 11% decline in livestock productivity by 2050 [1]. AMR is defined as the resistance of microorganisms to clinically relevant antimicrobial medications at standard doses [2]. Furthermore, bacteria are called multidrug-resistant (MDR) when they are resistant to at least three classes of antimicrobials [3]. One of the most significant consequences of the indiscriminate use of antimicrobials, which is known as the “Silent Pandemic”, may be the global spread of MDR strains [4]. Since the discovery of the first antimicrobial therapy, resistance to antimicrobials has been considered a natural process in which microbes evolve to resist the effects of drugs [5]. The combination of the overuse of antimicrobials leading to reduced treatment efficacy and the lack of new antimicrobial development to combat these new superbugs has progressively increased the risks of AMR in recent years [6].

AMR is a significant and prevalent issue in food-producing animals but receives insufficient attention. In general, antimicrobials are used in the dairy industry to treat diseases like mastitis and for both therapeutic and preventive purposes [7]. The use of sub-therapeutic doses of antimicrobials for prophylactic and growth promotion is a risk for AMR development in animal production environments, including the dairy industry. By 2030, the use of antimicrobials (AMU) in food-producing animals will increase by more than 67% to meet this demand [8]. The primary concern about AMR in animals is that resistant strains of bacteria could spread zoonotically from animals to humans [9]. Humans may be exposed to resistant strains and genes if they consume contaminated food, such as contaminated meat, unpasteurised milk, and milk products, or if resistant strains and genes spread through the environment, such as animal waste and runoff water from agricultural sites, or via direct animal contact [10,11]. Milk and milk products can harbour diverse microorganisms and serve as significant sources of pathogens that propagate through food. In Bangladesh, widespread consumption of raw milk, often produced without strict sanitary controls, raises concerns as antibiotic use in livestock can promote resistant strains like *E. coli*, posing significant health risks to consumers. Milk can become contaminated with foodborne pathogens like *E. coli* through direct contact with infected sources on a dairy farm or the introduction of udder debris (bovine faeces, environmental contaminants) from an infected animal, posing a risk of infection to humans [12].

The emergence of antibiotic-resistant *E. coli* presents a major global health threat, posing significant challenges to veterinary care, public health, and dairy cattle producers by complicating treatment efforts [13]. Various AMR genes are responsible for antimicrobial resistance, which bacteria can readily acquire through horizontal gene transfer mechanisms [14,15,16]. Over time, a key risk is the accumulation of resistance genes that will confer a broad range of AMR phenotypes, including MDR [17]. Beyond the foodborne risk, the spread of MDR *E. coli* is a public health concern because it poses a risk to farm workers and other people who encounter animals [18]. In *E. coli*, resistance to a broad spectrum of β-lactam antibiotics is often spread via horizontal gene transfer of extended-spectrum beta-lactamase (ESBL) genes, with ESBL-producing strains more likely to exhibit multidrug resistance, making infections more difficult to treat [19]. Many ESBL genes, such as *bla*_CTX-M_, *bla*_TEM_, P*amp*C, *bla*_OXA_, *bla*_CMY_, and *bla*_ACC1_, have also been found in faecal samples from pigs, cattle, chickens, and sheep [20,21]. This is because a lack of knowledge and uncontrolled access to medicines can lead to increased use and more inappropriate use of antimicrobials [22]. The utilisation of antimicrobials in Bangladesh’s livestock industry is entirely irrational, which makes the spread of AMR more likely [23]. AMR problems can also emerge in developing countries like Bangladesh due to the lack of adequate healthcare infrastructure [24]. The current investigation aims to ascertain the pattern and prevalence of antimicrobial resistance in *E. coli* strains isolated from raw milk sources in Bangladesh. The key research questions are to determine the prevalence of *E. coli* in raw milk, analyse their resistance profiles against key antimicrobials of public health relevance, and identify associated resistance genes using genetic analysis. This study aims to provide valuable insights into the public health risks of antimicrobial resistance transmission from dairy milk to humans, particularly within the context of a developing country.

## 2. Materials and Methods

### 2.1. Study Design and Sample Collection

**Figure 1:**
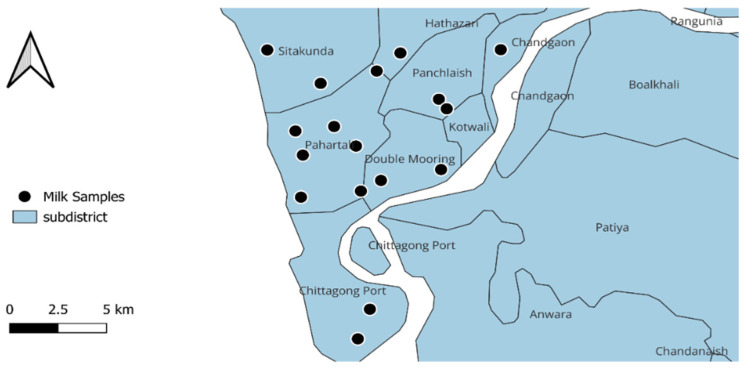
Geographical locations of the farms randomly selected for sampling in this study.

This study was conducted between September 2021 and August 2022 in the Chattogram metropolitan area (CMA) within the Chattogram district of Bangladesh. A total of 18 large-scale dairy farms hosting ≥50 dairy cows from seven locations, including Patenga, Akbershah, Dewanhut, Foys Lake, Sadarghat, Pahartali, and Wireless Area, were selected randomly for sample collection. These farm locations are illustrated in Figure 1. From each farm, 25 raw milk samples were collected from randomly selected cows after cleansing the udders and teat-ends with cotton soaked in 70% isopropanol to ensure aseptic condition. This study did not include cows with a recent treatment history and any active diseases, including mastitis. A total of 450 raw milk samples were collected in separate Falcon tubes using an aseptic technique.

### 2.2. Sample Preparation

After collection, samples were transferred to the Department of Physiology, Biochemistry and Pharmacology (DPBP), CVASU, for further investigation whilst maintaining a cold chain. For primary enrichment, samples were diluted with buffered peptone water (BPW) (HIMEDA, Mumbai, India), maintaining a ratio of 9:1 (BPW: cow milk sample), and incubated at 37 °C overnight.

### 2.3. Phenotypic Isolation and Identification of E. coli

To isolate *E. coli*, a loopful of enriched broth (BPW) was inoculated onto MacConkey agar (HIMEDIA, Mumbai, India) and incubated at 37 °C for 24 h. Suspected colonies were inoculated onto Eosin Methylene Blue (EMB) agar (HIMEDIA, Mumbai, India) and incubated at 37 °C for 24 h for biochemical confirmation. Confirmed colonies were then inoculated onto blood agar (HIMEDIA, Mumbai, India) and incubated at 37 °C for 24 h. All phenotypically confirmed *E. coli* isolates were incubated overnight at 37 °C in brain heart infusion (BHI) broth (HIMEDIA, Mumbai, India). After incubation, 700 µL of BHI broth was added to 300 µL of 15% glycerol in an Eppendorf tube for each isolate and stored at −80 °C for further investigations.

### 2.4. Molecular Confirmation of E. coli

All phenotypically positive *E. coli* isolates were subjected to molecular identification by multiplex PCR using species-specific primers targeting *uid*A and *usp*A genes. The genomic DNA was extracted following the crude boiling method [25]. The multiplex PCR assay was conducted using the following primes: *usp*A (F) CCGATACGCTGCCAATCAGT; *usp*A (R) ACGCAGACCGTAGGCCAGAT; *uid*A (F)TATGGAATTTCGCCGATTTT; and *uid*A (R) TGTTTGCCTCCCTGCTGCGG, maintaining the initial denaturation at 94 °C for 5 min and final extension at 72 °C for 10 min with the 35 cycles of denaturation at 94 °C for 10 s, annealing at 52.2 °C for 10 s, and extension at 72 °C for 1 min [26]. All PCR reactions were performed on a thermal cycler (DLAB Scientific Inc., Alhambra, CA, USA) with a final volume of 25 µL containing 12.5 µL DreamTaq 2X master mix (Thermofisher Scientific, Waltham, MA, USA), 1 µL forward and reverse primer (10 pmol/µL), 2 µL template DNA, and ~8.5 µL Nuclease-free water, in the research lab under the DPBP, CVASU. All the amplified PCR products were screened by electrophoresis with a 1.5% agarose gel (MP Biomedicals, Santa Ana, CA, USA) for 25 min at 120 V in 1x TAE buffer and visualised using ethidium bromide (Sigma Aldrich, Burlington, MA, USA) on a gel documentation system (UVP UVsolo touch-Analytik Jena AG, Thermo Fisher Scientific, Waltham, MA, USA). ATCC 25922 *E. coli* strains were used as a positive control, and nuclease-free water (NFW) as a negative control.

### 2.5. Phenotypic Antimicrobial Resistance Profiles

All *E. coli* isolates were screened for antimicrobial susceptibility against a panel of antimicrobials using the Kirby–Bauer disc diffusion method [27]. A total of 17 antimicrobials representing seven different antimicrobial groups (penicillins, cephalosporins, phenicols, tetracyclines, aminoglycosides, fluoroquinolones, and sulfonamides) with public health significance were selected. The following antimicrobial agents were used: AMP: ampicillin (10 µg); AUG: amoxicillin–clavulanic acid (30 µg); KF: cephalothin (30 µg); CL: cephalexin (30 µg); FOX: cefoxitin (30 µg); CTX: cefotaxime (30 µg); CAZ: ceftazidime (30 µg); FFC: Florfenicol (30 µg); TE: tetracycline (30 µg); DO: doxycycline (30 µg); CN: gentamicin (10 µg); N: neomycin (30 µg); CIP: ciprofloxacin (5 µg); LEV: levofloxacin (5 µg); ENR: enrofloxacin (5 µg); NOR: norfloxacin (10 µg); and SXT: sulfamethoxazole–trimethoprim (23.75 + 1.25 µg) (Oxoid Ltd., Hampshire, UK). The bacterial suspension was adjusted to the turbidity of 0.5 McFarland standard (equivalent to growth of 1–2 × 10^8^ CFU/mL) and streaked over the entire dry surface of Mueller Hinton agar (Oxoid Ltd.^®^, pH 7.3 ± 0.1) three times, rotating the plate approximately at 60 degrees by a sterile swab stick. Following incubation, the diameter of the disc and the extent of the inhibition zone (measured in millimetres) were recorded, and the results were interpreted according to the Clinical Laboratory Standards Institute’s guidelines [28]. Isolates that were resistant to more than 3 antimicrobial classes were termed multidrug-resistant (MDR) [29]. The multiple antibiotic resistance (MAR) index was estimated using the formula described previously by Algammal et al. [30].

### 2.6. Detection of AMR Genes

A total of 15 AMR genes were screened in this study, including those conferring ESBL-resistance (*bla*_TEM_, *bla*_SHV_, *bla*_CTX-M_, *bla*_OXA-1_, *bla*_OXA-2_, *bla*_CMY-1_, *bla*_CMY-2_, *bla*_ACC-1_, P*amp*C)_,_ sulfonamides resistance (*sul*-1, *sul*-2), and tetracycline resistance (*tet*A, *tet*B, *tet*C, *tet*D), by PCR. We selected those genes based on the WHO classification of higher public health risk and the commonly used antimicrobials in dairy practice in Bangladesh. The oligonucleotide primer sequences, annealing temperature, and amplicon size are shown in Table 1. The pan drug-susceptible *E. coli* ATCC 25922 strain was used as a negative control in each PCR to detect AMR genes.

### 2.7. Statistical Analysis

All data were recorded and organised in Microsoft Excel 2019 for statistical analysis. The data were then analysed in STATA/IC-15 (Stata Corp, 4905 Lakeway Drive, College Station, TX, USA) to estimate the prevalence and 95% confidence intervals (CI). The correlation coefficients among antimicrobials, phenotypic AMR, and resistance genes were calculated and illustrated using R software (version 4.4.1; https://www.r-project.org/; accessed on 20 October 2024) with the ggplot2 (ggcorrplot version 0.1.4.1) package. The geographical map was constructed using ArcGIS version 10.8.

## 3. Results

### 3.1. Prevalence of E. coli

A total of 450 milk samples from 18 CMA farms were investigated in this study. The demographic information for this study is summarised in Supplementary Table S1, which lists the variables, sample categories, and sample sizes for each category. The detection rate of *E. coli* was highest on Farm 18 (64%, 95% CI: 42.52–82.03) and lowest at 12% (95% CI: 2.55–31.22) on both Farms 7 and 10 (Supplementary Table S1). A total of 134 isolates (29.77%; 95% CI: 25.59–34.24) were confirmed as *E. coli* from raw milk samples.

### 3.2. AMR Profiles of Isolated E. coli

**Figure 2:**
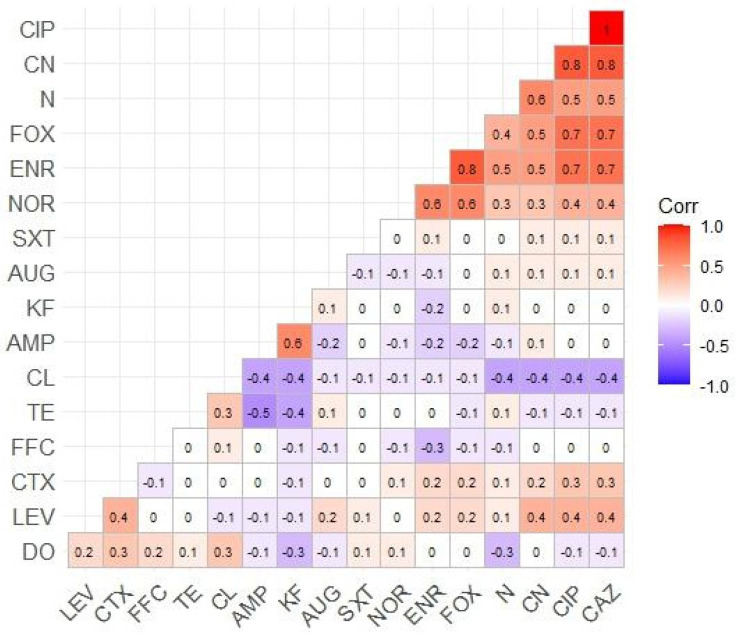
Heatmap showing the correlation coefficient among antimicrobials tested in this study.

The AST revealed that four beta-lactam antimicrobials, including ampicillin, amoxicillin–clavulanic acid, cephalothin, and cephalexin, showed the highest rates of resistance (69.40%), followed by sulfamethoxazole–trimethoprim (68.65%) and florfenicol (55.97%). In contrast, the lowest resistance rate was observed in the fluoroquinolones, ciprofloxacin (23.88%), levofloxacin (23.88%), and norfloxacin (21.64%), but not enrofloxacin (41.79%). The tetracyclines exhibited a similarly high resistance rate (51.49%). ESBL antibiotics, such as cefotaxime and ceftazidime, had similar resistance rates to each other (23.88%), except for cefoxitin (51.49%). Resistance rates for aminoglycosides, such as gentamicin and neomycin, were 51.49% and 32.84%, respectively. The AMR profiles are shown in Table 2. Correlation coefficients among antimicrobials tested in this study displayed a significant level of correlation between resistance rates for many antimicrobials, e.g., CIP and CAZ (*r* = 1); CIP, CAZ, and CN (*r* = 0.8); ENR and FOX (*r* = 0.8); CIP, CAZ, FOX, and ENR (*r* = 0.7); N and CN (*r* = 0.6), as illustrated in Figure 2.

### 3.3. Phenotypic MDR Patterns of E. coli Isolates

**Figure 3:**
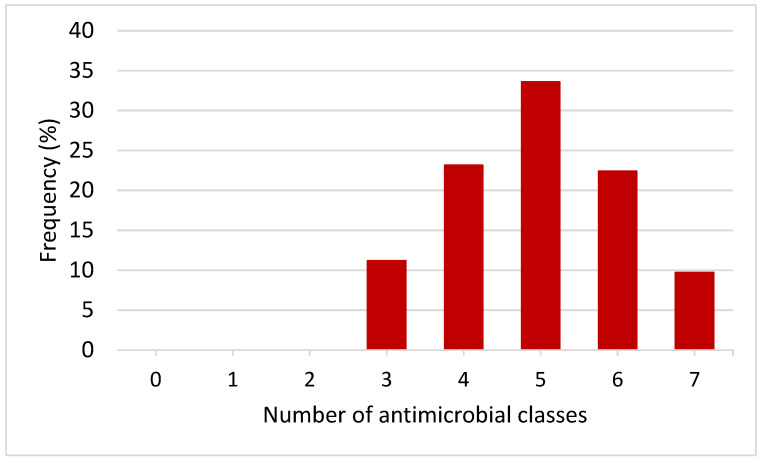
MDR profiles of *E. coli* isolates from raw cow milk.

All the *E. coli* isolates from raw cow milk were classified as MDR (resistant to at least three or more antimicrobial classes) (Figure 3). The MDR patterns varied between isolates, with most showing unique resistance profiles. Only 2.24% (3/134) of the isolates exhibited the same resistance pattern (LEV, SXT, DO, AMP, AUG, KF, CL, FFC). The MDR patterns are illustrated in Table 3. The MAR index in this study ranged from 0.24 to 0.82 (Table 3).

### 3.4. Distribution of AMR Genes

**Figure 4:**
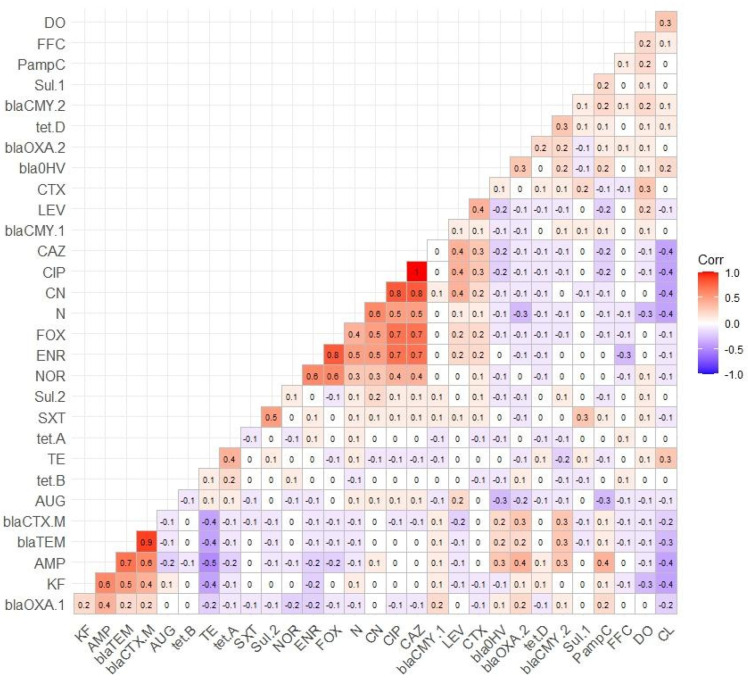
The correlation coefficient between phenotypic AMR and resistance genes.

A total of nine ESBL genes were tested. Among them, the prevalence of the *bla*_TEM_ gene was the highest (74.19%), followed by *bla*_CTX-M_ (69.89%), *bla*_OXA-2_ (40.86%), P*amp*C (37.63%), and *bla*_OXA-1_ (33.33%). In contrast, the prevalence of the *bla*_CMY-1_ gene was lower (6.45%), while *bla*_ACC-1_ was absent. Among the tetracycline resistance genes tested, the prevalence of the *tet*A gene was highest (30.43%), followed by *tet*B (5.8%) and *tet*D (2.9%), and the *tet*C gene was absent. Similarly, the prevalence of sulfonamide resistance genes, *sul*-1 and *sul*-2, was 25% and 55.43%, respectively. The prevalence of antimicrobial resistance genes is shown in Table 4. A positive correlation was observed between phenotypic AMR and resistance genes, including *AMP* and *bla*_TEM_ (*r* = 0.7), *AMP* and *bla*_CTX-*a*_ (*r* = 0.6), *KF* and *bla*_TEM_ (*r* = 0.5), *KF* and *bla*_CTX-M_ (*r* = 0.4), *SXT* and *sul*-2 (*r* = 0.5), *TE* and *tet*A (*r* = 0.4), *AMP* and P*amp*C (*r* = 0.4), as illustrated in Figure 4.

## 4. Discussion

The current study revealed a high prevalence of *E. coli* in the milk of Bangladeshi farms collected from various dairy cattle, with the isolates frequently displaying resistance to multiple antimicrobial classes. At the farm level, the prevalence of *E. coli* varied between 12 to 64%. In this study, the overall prevalence of *E. coli* in farm milk is approximately 30%, less than the 42% prevalence reported in Iran by Vahedi et al. [34]. The variation in the prevalence of *E. coli* in the present study might be due to variations in hygiene and managemental practices in different farms. Another study reported the same 42% prevalence of *E. coli* in milk in Ethiopia [35]. Studies indicated that the prevalence of *E. coli* contamination in raw milk in Bangladesh is notably high, ranging from 50% to 92%, often linked to poor hygiene practices during milking and handling [36]. Global contamination rates vary significantly, with lower incidences in developed countries, such as the US, where *E. coli* contamination in milk is generally below 10% due to stricter regulations and better sanitation [37]. This disparity suggests that faecal contamination and hygiene standards are poorer in Bangladesh than in many other regions. In this study, 17 antimicrobials were tested using the AST, and 15 resistance genes were tested against those antimicrobials. The AST of the isolates revealed that *E. coli* resistant to ampicillin, amoxicillin–clavulanic acid, cephalothin, and cephalexin was the most prevalent, followed by sulfamethoxazole–trimethoprim and florfenicol. A recent study showed 15% resistance to sulfonamide and 3% resistance to trimethoprim [38]. Another one reported the prevalence of *E. coli* in calves from a dairy farm was 37.5% [39]. In contrast, this study revealed that out of 92 sulfamethoxazole–trimethoprim isolates, 23 were resistant to the *sul*-1 gene, and 51 were resistant to the *sul*-2 gene.

*E. coli* antimicrobial resistance for the isolates was found to be conferred by a wide range of resistance genes. These genes include the tet genes (*tet*A, *tet*B, *tet*C, *tet*D), gene for Tetracycline resistance, the *bla*_TEM_, *bla*_SHV_, P*amp*C, *bla*_OXA_, *bla*_ACC_, *bla*_CMY_, *bla*_CTX-M_ genes for Ampicillin, and *Sul*-1, *Sul*-2 genes for Trimethoprim-sulfamethoxazole [14,15,16]. Evidence shows that AMR patterns in dairy farms in Bangladesh reflect the high usage of specific antimicrobials, particularly β-lactams, commonly used in treating mastitis and other infections [40]. High rates of extended-spectrum β-lactamase (ESBL)-producing *E. coli* have been reported in dairy environments, likely due to the extensive use of β-lactam antibiotics [16]. This supports the hypothesis that increased antimicrobial use correlates with higher resistance rates. This pattern aligns with global observations that frequent use of antimicrobials drives the development of resistance in microbial populations [41]. In Bangladesh, certain antimicrobials, including fluoroquinolones and aminoglycosides, are not strictly regulated for use in dairy cattle, despite their potential to contribute to AMR, unlike in countries like Australia [42], where their use in food-producing animals is banned. The relevance to the current study is significant, as the continued use of these drugs in Bangladesh likely contributes to the high resistance rates observed, particularly for classes like fluoroquinolones.

A previous study reported that 13.4% of *E. coli* isolated from milk were resistant to tetracycline [43], but in this study, the resistance was higher (51%). Among 69 tetracycline-resistant isolates, *tet*A was the most frequently detected gene, accounting for 21 (86.5%), while *tet*B was detected in only 4 (8.1%) isolates. Only two resistant genes for *tet*C and *tet*D were detected. According to [14], only 0.4% of faecal samples were positive for ESBL-producing *E. coli* isolated from a lactating bovine, while 6.5% of the farm environment samples were positive. Hassan [16] reported that 62.50% of milk samples contained ESBL *E. coli* with the gene combination *bla*_TEM_ + *bla*_CTX-M_. Correlations between resistance rates for different antimicrobials suggest co-selection, where resistance to one antimicrobial may confer resistance to others due to shared resistance mechanisms, such as plasmids carrying multiple resistance genes. This phenomenon is exacerbated by polypharma practices, where the overuse of multiple antimicrobials in dairy farming can lead to the selection of multidrug-resistant bacteria. Studies have shown that the widespread use of various antimicrobials in regions like Bangladesh promotes co-selection, driving the persistence of multidrug-resistant organisms in agricultural environments [44,45].

The World Health Organization (WHO) categorised antimicrobials into three groups to mitigate the situation: access; monitor; and reserve groups [46]. Access group antimicrobials are available to be prescribed to patients by physicians. If this group fails due to resistant genes in organisms, it is recommended that the patient be monitored in a separate group [47]. Reserve categories of antimicrobials are for future use if others become resistant. Scientific knowledge and evidence are required to mitigate AMR issues before they become widespread crises. AMR is among the deadliest threats to human and animal health. To alleviate the AMR health threat before it manifests in large-scale medical emergencies, it is necessary to identify risks and appropriate mitigation strategies based on scientific evidence and knowledge. Our finding showed that most isolates in this study were MDR, suggesting a much higher prevalence of MDR in livestock-associated *E. coli* in dairy farms. This poses serious concerns for both animal and public health, as MDR bacteria reduce treatment options for infections in dairy cattle, leading to potential economic losses and increased animal suffering. Additionally, the spread of MDR pathogens from animals to humans through direct contact or consuming contaminated dairy products represents a significant public health risk, particularly in regions like Bangladesh.

## 5. Limitations

This study’s scope is confined to farms within the Chattogram metropolitan area of Bangladesh, limiting the generalisability of the findings to be applied to other regions or dairy farming practices nationally. The focus on *E. coli* alone may overlook the presence of other critical antimicrobial-resistant bacteria in raw milk that could pose a public health risk. However, *E. coli* is a widely used indicator organism in food microbiology, such that levels of resistance in *E. coli* are reflective of general antimicrobial selection pressures and levels of resistance amongst other enteric microbes, including foodborne pathogens.

## 6. Conclusions

This study reveals a high prevalence of AMR and MDR *E. coli* in raw cow milk from dairy farms in the Chattogram metropolitan area, with significant resistance to widely used antimicrobials such as ampicillin and amoxicillin–clavulanic acid. The findings show a strong link between resistance patterns and the presence of resistance genes, particularly *bla*_TEM_, highlighting the risk of resistant bacteria transmission to humans via the dairy food chain, a serious public health concern. Urgent strategic interventions, including prudent antimicrobial use in dairy farming and enhanced AMR surveillance, are essential to control AMR spread in the dairy sector and protect public health.

## Figures and Tables

**Table 1:** The oligonucleotide primer sequences for the detection of AMR genes in *E. coli* isolated from raw dairy milk.

Antimicrobial Agents	Target Gene	Primer NAME	Primer Sequence (5′-3′)	Annealing Temp.	Amplicon Size (bp)	References
Tetracyclines	*tet*A	*tet*A-F	CGCCTTTCCTTTGGGTTCTCTATATC	55 °C	182	[31]
		*tet*A-R	CAGCCCACCGAGCACAGG			
	*tet*B	*tet*B-F	GCCAGTCTTGCCAACGTTAT		975	
		*tet*B-R	ATAACACCGG TTGCATTGGT			
	*tet*C	*tet*C-F	TTCAACCCAGTCAGCTCCTT		560	
		*tet*C-R	GGGAGGCAGACAAGGTATAGG			
	*tet*D	*tet*D-F	GAGCGTACCGCCTGGTTC		780	
		*tet*D-R	TCTGATCAGCAGACAGATTGC			
Sulphonamides	*sul*-1	*sul*-1-F	CGGCGTGGGCTACCTGAACG	68 °C	779	[32]
		*sul*-1-R	GCCGATCGCGTGAAGTTCCG			
	*sul*-2	*sul*-2-F	CCTGTTTCGTCCGACACAGA	66 °C	721	
		*sul*-2-R	GAAGCGCAGCCGCAATTCAT			
ESBLs	*bla*_TEM_	*bla*_TEM_-F	ATAAAATTCTTGAAGACGAAA	54 °C	964	[33]
		*bla*_TEM_-R	GACAGTTACCAATGCTTAATC			
	*bla*_SHV_	*bla*_SHV_-F	GCTTTCCCATGATGAGCACC	50 °C	854	
		*bla*_SHV_-R	AGGCGGGTGACGTTGTCGC			
	P*amp*C	P*amp*C-F	GTGAATACAGAGCCAGACGC	50 °C	343	
		P*amp*C-R	GTTGTTTCCGGGTGATGC			
	*bla*_OXA-1_	*bla*_OXA-1_-R	GTGTGTTTAGAATGGTGATCGCATT	62 °C	820	
		*bla*_OXA-1_-R	GTGTGTTTAGAATGGTGATCGCATT			
	*bla*_OXA-2_	*bla*_OXA-2_-F	ACGATAGTTGTGGCAGACGAAC	62 °C	602	
		*bla*_OXA-2_-R	ATYCTGTTTGGCGTATCRATATTC			
	*bla*_CTX-M_	*bla*_CTX-M_-F	ATGTGCAGYACCAGTAARGTKATGGC	60 °C	593	
		*bla*_CTX-M_-F	TGGGTRAARTARGTSACCAGAAYCAGCGG			
	*bla*_CMY-1_	*bla*_CMY-1_-F	GTGGTGGATGCCAGCATCC	58 °C	915	
		*bla*_CMY-1_-R	GGTCGAGCCGGTCTTGTTGAA			
	*bla*_CMY-2_	*bla*_CMY-2_-F	GCACTTAGCCACCTATACGGCAG	58 °C	758	
		*bla*_CMY-2_-R	GCTTTTCAAGAATGCGCCAGG			
	*bla*_ACC-1_	*bla*_ACC-1_-F	ATYCTGTTTGGCGTATCRATATTC	53 °C	818	
		*bla*_ACC-1_-R	AGCCTCAGCAGCCGGTTAC			

**Table 2:** Phenotypic antimicrobial susceptibility test profiles of *E. coli* isolates.

Antimicrobial Groups	Antimicrobial Agents	Susceptible (S) N (%)	Intermediate (I) N (%)	Resistant (R) N (%)
Penicillins	AMP (10 µg)	36 (26.86)	5 (3.73)	93 (69.40)
	AUG (30 µg)	36 (26.86)	5 (3.73)	93 (69.40)
Cephalosporins	KF (30 µg)	40 (29.85)	1 (0.75)	93 (69.40)
	CL (30 µg)	41 (30.59)	0 (0)	93 (69.40)
	FOX (30 µg)	34 (25.37)	44 (32.84)	56 (41.79)
	CTX (30 µg)	53 (39.55)	49 (36.57)	32 (23.88)
	CAZ (30 µg)	53 (39.55)	49 (36.57)	32 (23.88)
Phenicols	FFC (30 µg)	49 (36.56)	10 (7.46)	75 (55.97)
Tetracyclines	TE (30 µg)	40 (29.85)	25 (18.66)	69 (51.49)
	DO (30 µg)	52 (38.81)	13 (9.70)	69 (51.49)
Aminoglycosides	CN (30 µg)	45 (33.58)	45 (33.58)	44 (32.84)
	N (30 µg)	25 (18.65)	40 (29.85)	69 (51.49)
Fluoroquinolones	CIP (5 µg)	53 (39.55)	49 (36.57)	32 (23.88)
	LEV (5 µg)	80 (59.70)	22 (16.41)	32 (23.88)
	ENR (5 µg)	34 (25.37)	44 (32.83)	56 (41.79)
	NOR (10 µg)	85 (63.43)	20 (14.92)	29 (21.64)
Sulfonamides	SXT (23.75 + 1.25 µg)	40 (29.85)	2 (1.5)	92 (68.65)

**Table 3:** The phenotypic MDR patterns of *E. coli* isolates in this study.

Phenotypic Multidrug Resistance Patterns	No. of Isolates (%)	MAR Index
SXT, DO, AUG, KF, CL, FFC	1 (0.75)	0.35
DO, AUG, CL, FFC	1 (0.75)	0.24
ENR, NOR, SXT, TE, DO, AMP, AUG, KF, CL, FOX	1 (0.75)	0.59
N, ENR, SXT, AMP, CL	1 (0.75)	0.29
AUG, KF, CL, FOX, FFC	2 (1.49)	0.29
SXT, KF, CL, FFC	1 (0.75)	0.24
SXT, DO, AMP, KF, FFC	1 (0.75)	0.29
ENR, NOR, SXT, AMP, CL, FOX	1 (0.75)	0.35
TE, DO, AMP, AUG, KF, CL, FFC	1 (0.75)	0.41
DO, AMP, CL, FOX, FFC	1 (0.75)	0.29
ENR, NOR, DO, CL, FOX	1 (0.75)	0.29
TE, DO, AMP, KF, CL, FFC	1 (0.75)	0.35
TE, DO, AMP, CL, FFC	1 (0.75)	0.29
ENR, NOR, TE, DO, AMP, CL, FOX	1 (0.75)	0.41
N, ENR, TE, DO, AMP, CL, FFC	1 (0.75)	0.41
CN, N, ENR, NOR, DO, AMP, KF, CL, FOX, FFC	1 (0.75)	0.59
N, AMP, AUG, KF, CL, FOX, CTX, FFC	1 (0.75)	0.47
SXT, DO, AMP, KF, CL, CTX, FFC	1 (0.75)	0.41
ENR, NOR, SXT, DO, AMP, KF, CL, FOX, CTX	1 (0.75)	0.53
N, ENR, SXT, TE, AMP, KF, CL, CTX	1 (0.75)	0.47
AMP, AUG, KF, CL, FFC	1 (0.75)	0.29
SXT, AMP, KF, CL, FFC	1 (0.75)	0.29
LEV, SXT, TE, DO, AMP, KF, CL, CTX, FFC	1 (0.75)	0.53
ENR, NOR, DO, AMP, KF, CL, FOX, FFC	1 (0.75)	0.47
ENR, SXT, DO, AMP, KF, CL, FOX, FFC	1 (0.75)	0.47
SXT, TE, AMP, AUG, KF, CL, FFC	1 (0.75)	0.41
TE, AMP, KF, CL, FFC	1 (0.75)	0.29
N, ENR, NOR, SXT, TE, DO, KF, CL, FOX	1 (0.75)	0.53
CN, N, SXT, TE, AMP, KF, CL	2 (1.49)	0.41
AMP, AUG, KF, CL, FOX, FFC	1 (0.75)	0.35
SXT, AMP, AUG, KF, CL, FOX, FFC	1 (0.75)	0.41
SXT, DO, AMP, KF, CL, FFC	2 (1.49)	0.35
ENR, NOR, SXT, DO, AMP, CL, FOX, CTX, FFC	1 (0.75)	0.53
ENR, SXT, DO, AMP, CL, CTX	1 (0.75)	0.35
SXT, TE, DO, AMP, CL, CTX	1 (0.75)	0.35
CN, N, LEV, SXT, DO, AMP, AUG, KF, CL, CTX, FFC	1 (0.75)	0.65
CN, N, CIP, LEV, ENR, SXT, DO, AMP, AUG, KF, FOX, CTX, CAZ, FFC	1 (0.75)	0.82
SXT, DO, AMP, AUG, KF, CL	2 (1.49)	0.35
CN, N, CIP, LEV, ENR, NOR, SXT, DO, AMP, AUG, KF, FOX, CTX, CAZ	1 (0.75)	0.82
SXT, TE, DO, AMP, AUG, KF, CL, CTX	1 (0.75)	0.47
CN, N, SXT, TE, DO, AMP, AUG, CL, FFC	1 (0.75)	0.53
CN, N, CIP, LEV, ENR, NOR, TE, DO, AUG, CL, FOX, CTX, CAZ, FFC	1 (0.75)	0.82
CN, N, CIP, ENR, NOR, SXT, TE, DO, AUG, KF, FOX, CTX, CAZ, FFC	1 (0.75)	0.82
CN, N, DO, AMP, AUG, KF, CL, CTX	1 (0.75)	0.47
CN, N, CIP, ENR, SXT, DO, AMP, AUG, KF, FOX, CTX, CAZ, FFC	1 (0.75)	0.76
LEV, SXT, DO, AMP, AUG, KF, CL, CTX	1 (0.75)	0.47
CN, N, CIP, ENR, NOR, SXT, DO, AMP, AUG, KF, FOX, CTX, CAZ	1 (0.75)	0.76
LEV, ENR, SXT, TE, DO, AUG, CL, FOX, CTX	1 (0.75)	0.53
ENR, TE, DO, AUG, CL, CTX	1 (0.75)	0.35
CN, N, CIP, SXT, LEV, ENR, NOR, TE, DO, AUG, CL, FOX, CTX, CAZ, FFC	1 (0.75)	0.88
LEV, SXT, TE, DO, AUG, CL, CTX	1 (0.75)	0.41
CN, N, TE, AMP, AUG, KF, FFC	1 (0.75)	0.41
N, ENR, SXT, TE, DO, AUG, KF, CL, FOX	2 (1.49)	0.53
N, SXT, TE, AMP, AUG, KF	1 (0.75)	0.35
LEV, SXT, TE, DO, AMP, AUG, KF, CL, CTX	1 (0.75)	0.53
TE, AMP, AUG, KF	2 (1.49)	0.24
SXT, AMP, AUG, KF	2 (1.49)	0.24
LEV, SXT, AMP, AUG, KF	1 (0.75)	0.29
N, ENR, SXT, TE, AUG, KF, CL, FOX	1 (0.75)	0.47
CN, N, SXT, AMP, AUG, KF	1 (0.75)	0.35
CN, N, CIP, LEV, ENR, NOR, TE, DO, AUG, CL, FOX, CTX, CAZ	1 (0.75)	0.76
LEV, TE, DO, AUF, CL, CTX, FFC	1 (0.75)	0.41
N, TE, DO, AUG, CL, FFC	1 (0.75)	0.35
CN, N, CIP, LEV, ENR, AMP, AUG, KF, FOX, CAZ, FFC	1 (0.75)	0.65
LEV, SXT, DO, AMP, AUG, KF, CL, FFC	3 (2.24)	0.47
CN, N, SXT, DO, AMP, AUG, KF, FFC	1 (0.75)	0.47
CN, N, CIP, LEV, ENR, NOR, TE, DO, AUG, CL, FOX, CAZ, FFC	1 (0.75)	0.76
CN, N, CIP, ENR, SXT, TE, DO, AUG, CL, FOX, CAZ	1 (0.75)	0.65
N, SXT, AMP, KF, FFC	1 (0.75)	0.29
CN, LEV, SXT, TE, DO, AUG, CL, FFC	1 (0.75)	0.47
SXT, TE, DO, AMP, AUG, KF, CL, FFC	2 (1.49)	0.47
CN, N, CIP, LEV, ENR, NOR, SXT, AMP, AUG, KF, FOX, CAZ	1 (0.75)	0.71
CN, N, CIP, ENR, SXT, TE, AUG, CL, FOX, CAZ	1 (0.75)	0.59
CN, N, LEV, DO, AMP, AUG, KF, CL	1 (0.75)	0.47
N, SXT, TE, AUG, CL, FFC	1 (0.75)	0.35
CN, N, CIP, ENR, SXT, TE, DO, AUG, CL, FOX, CAZ, FFC	1 (0.75)	0.71
SXT, DO, AMP, AUG, KF, CL, FFC	2 (1.49)	0.41
N, SXT, AMP, AUG, KF, FFC	2 (1.49)	0.35
LEV, SXT, TE, DO, CL, FFC	1 (0.75)	0.35
N, SXT, TE, CL, FFC	1 (0.75)	0.29
SXT, TE, DO, CL, FFC	2 (1.49)	0.29
CN, N, CIP, LEV, ENR, NOR, SXT, AMP, KF, FOX, CAZ, FFC	1 (0.75)	0.71
CN, N, CIP, ENR, SXT, AMP, KF, FOX, CAZ	1 (0.75)	0.53
CN, N, CIP, ENR, NOR, SXT, AMP, KF, FOX, CAZ	1 (0.75)	0.59
SXT, TE, CL, FFC	1 (0.75)	0.24
SXT, TE, DO, AUG, CL, FFC	1 (0.75)	0.29
CN, N, CIP, LEV, ENR, SXT, AMP, AUG, KF, FOX, CTX, CAZ, FFC	1 (0.75)	0.76
N, SXT, TE, DO, AMP, AUG, KF, FFC	1 (0.75)	0.47
CN, N, CIP, ENR, NOR, SXT, TE, AMP, AUG, KF, FOX, CAZ, FFC	1 (0.75)	0.76
CN, N, CIP, LEV, ENR, NOR, SXT, TE, DO, AUG, CL, FOX, CTX, CAZ	1 (0.75)	0.82
N, AMP, AUG, KF	2 (1.49)	0.24
CN, N, CIP, ENR, NOR, SXT, AMP, AUG, KF, FOX, CAZ, FFC	1 (0.75)	0.71
N, SXT, TE, AMP, AUG, KF, FFC	1 (0.75)	0.41
CN, N, CIP, ENR, SXT, TE, AMP, AUG, FOX, CTX, CAZ, FFC	1 (0.75)	0.71
CN, N, CIP, ENR, NOR, SXT, AMP, AUG, KF, FOX, CAZ	1 (0.75)	0.65
CN, N, CIP, LEV, ENR, SXT, TE, AUG, CL, FOX, CAZ	1 (0.75)	0.65
TE, DO, AUG, CL, FFC	1 (0.75)	0.29
N, TE, AUG, CL, FFC	1 (0.75)	0.29
CN, N, CIP, ENR, NOR, AMP, AUG, KF, FOX, CAZ, FFC	1 (0.75)	0.65
CN, N, CIP, LEV, ENR, AMP, AUG, KF, FOX, CAZ	1 (0.75)	0.59
SXT, AMP, AUG, KF, CL	2 (1.49)	0.29
SXT, TE, AMP, AUG, KF, CL	1 (0.75)	0.35
N, ENR, NOR, SXT, TE, AUG, KF, CL, FOX	1 (0.75)	0.53
ENR, SXT, TE, AUG, CL, FOX	1 (0.75)	0.35
N, LEV, ENR, SXT, TE, AUG, CL	1 (0.75)	0.41
SXT, TE, AUG, CL	1 (0.75)	0.41
N, ENR, NOR, TE, AUG, KF, CL, FOX	1 (0.75)	0.47
N, SXT, TE, AMP, KF	1 (0.75)	0.29
TE, AMP, AUG, KF, CL, FFC	2 (1.49)	0.35
CN, N, CIP, LEV, ENR, SXT, AMP, KF, FOX, CTX, CAZ	1 (0.75)	0.65
CN, N, CIP, ENR, NOR, SXT, DO, AMP, KF, FOX, CAZ, FFC	1 (0.75)	0.71
CN, N, AMP, KF, CL, CTX	1 (0.75)	0.35
CN, N, CIP, LEV, ENR, AMP, KF, FOX, CTX, CAZ	1 (0.75)	0.59
CN, N, CIP, LEV, ENR, TE, DO, AMP, AUG, KF, FOX, CTX, CAZ, FFC	1 (0.75)	0.82
N, ENR, TE, AUG, KF, CL, FOX	1 (0.75)	0.41
N, TE, AMP, AUG, KF	1 (0.75)	0.29

Note: AMP = ampicillin; AUG = amoxicillin–clavulanic acid; KF = cephalothin; CL: cephalexin; FOX = cefoxitin; CTX = cefotaxime; CAZ = ceftazidime; FFC = Florfenicol; TE = tetracycline; DO = doxycycline; CN = gentamicin; N = neomycin; CIP = ciprofloxacin; LEV = levofloxacin; ENR = enrofloxacin; NOR= norfloxacin; SXT: sulfamethoxazole–trimethoprim; and MAR= multiple antibiotic resistance.

**Table 4:** Prevalence of antimicrobial resistance genes detected in *E. coli* isolates.

Resistance Genes	No. of Resistance Genes Present	Number of Phenotypic-Resistant Isolates (n = 134)	Resistance Gene, %, 95%CI
*tet*A	21	69	30.43 (20.80–42.13)
*tet*B	4	69	5.8 (1.85–14.40)
*tet*D	2	69	2.9 (0.2–10.57)
*sul*-1	23	92	25 (17.22–34.78)
*sul*-2	51	92	55.43 (45.26–65.17)
*bla*_TEM_	69	93	74.19 (64.42–82.05)
*bla*_SHV_	17	93	18.28 (11.64–27.43)
P*amp*C	35	93	37.63 (28.45–47.80)
*bla*_OXA-1_	31	93	33.33 (24.56–43.43)
*bla*_OXA-2_	38	93	40.86 (31.42–51.03)
*bla*_CTX-M_	65	93	69.89 (59.90–78.31)
*bla*_CMY-1_	6	93	6.45 (2.72–13.64)
*bla*_CMY-2_	16	93	17.20 (10.77–26.24)

## Data Availability

The data presented in this study are available in this article and Supplementary Material.

## Supplementary Materials

The following supporting information can be downloaded at: https://www.mdpi.com/article/10.3390/vetsci11120609/s1, Supplementary Table S1: Descriptive demography of the present study.
