# Supplementary material for: Epidemiology and Molecular Characterisation of Multidrug-Resistant Escherichia coli Isolated from Cow Milk
Source: Vet Sci. 2024 Nov 29;11(12):609. doi: 10.3390/vetsci11120609 (PMC11680316; doi:10.3390/vetsci11120609)
Supplement: Supplementary file 1 [file vetsci-11-00609-s001.zip › vetsci-3308085-supplementary.pdf]

Supplementary Table S1: Descriptive demography of the present study

| Variable | Category | Sample Size | Number of positive samples | Prevalence (%) (95% CI) |
|---|---|---|---|---|
| Sample type | Milk | 450 | 134 | 29.77 |
| Farm (F) | F1 | 25 | 5 | 20(6.83 – 40.7) |
| | F2 | 25 | 6 | 24 (9.36 – 45.13) |
| | F3 | 25 | 8 | 32 (14.95 – 53.5) |
| | F4 | 25 | 7 | 28 (12.07 – 49.39) |
| | F5 | 25 | 4 | 16 (4.54 – 36.08) |
| | F6 | 25 | 5 | 20 (6.83 – 40.7) |
| | F7 | 25 | 3 | 12(2.55 – 31.22) |
| | F8 | 25 | 5 | 20 (6.83 – 40.7) |
| | F9 | 25 | 9 | 36 (17.97 – 57.48) |
| | F10 | 25 | 3 | 12 (2.55 – 31.22) |
| | F11 | 25 | 6 | 24 (9.36 – 45.13) |
| | F12 | 25 | 10 | 40 (21.13 – 61.33) |
| | F13 | 25 | 8 | 32 (14.95 – 53.5) |
| | F14 | 25 | 10 | 40 (21.13– 61.33) |
| | F15 | 25 | 8 | 32 (14.95 – 53.5) |
| | F16 | 25 | 8 | 32 (14.95 – 53.5) |
| | F17 | 25 | 13 | 52 (31.31 – 72.2) |
| | F18 | 25 | 16 | 64 (42.52 – 82.03) |
